# Safety Level of Microorganism-Bearing Products Applied in Soil-Plant Systems

**DOI:** 10.3389/fpls.2022.862875

**Published:** 2022-04-28

**Authors:** Maria Vassileva, Stefano Mocali, Loredana Canfora, Eligio Malusá, Luis F. García del Moral, Vanessa Martos, Elena Flor-Peregrin, Nikolay Vassilev

**Affiliations:** ^1^Department of Chemical Engineering, Institute of Biotechnology, University of Granada, Granada, Spain; ^2^Council for Agricultural Research and Economics, Research Centre for Agriculture and Environment, Rome, Italy; ^3^Research Institute of Horticulture, Skierniewice, Poland; ^4^Council for Agricultural Research and Economics, Center for Viticulture and Enology, Conegliano, Italy; ^5^Department of Plant Physiology, University of Granada, Granada, Spain; ^6^Institute of Biotechnology, University of Granada, Granada, Spain

**Keywords:** microbial inoculants, pathogens, risks of contamination, safety measures and regulations, organic matter

## Abstract

The indiscriminate use of chemical fertilizers adversely affects ecological health and soil microbiota provoking loss of soil fertility and greater pathogen and pest presence in soil-plant systems, which further reduce the quality of food and human health. Therefore, the sustainability, circular economy, environmental safety of agricultural production, and health concerns made possible the practical realization of eco-friendly biotechnological approaches like organic matter amendments, biofertilizers, biopesticides, and reuse of agro-industrial wastes by applying novel and traditional methods and processes. However, the advancement in the field of Biotechnology/Agriculture is related to the safety of these microorganism-bearing products. While the existing regulations in this field are well-known and are applied in the preparation and application of waste organic matter and microbial inoculants, more attention should be paid to gene transfer, antibiotic resistance, contamination of the workers and environment in farms and biotech-plants, and microbiome changes. These risks should be carefully assessed, and new analytical tools and regulations should be applied to ensure safe and high-quality food and a healthy environment for people working in the field of bio-based soil amendments.

## Introduction

There is a generalized expert opinion that the major challenge facing agriculture is to increase crop productivity with a simultaneous reduction of environmentally damaging chemical fertilization. Intensive agricultural practices based on chemical fertilizers caused an adverse impact on autochthonous microbial communities (including plant beneficial microorganisms established in the rhizosphere) and microbial biodiversity with a simultaneous significant reduction of soil organic matter and mineral content (Mäder et al., 2002; Huang et al., 2019). With the aim of solving the problems arising from modern conventional agriculture and following the principles of sustainable agriculture and circular economy, the scientific efforts are focused on the development of less harmful strategies for stimulating plant growth and health by restoring soil fertility and microbial diversity. These strategies include attempts to close the nutrient cycle at farm level by maximizing reutilization of by-products and wastes, and restore beneficial plant-biological interactions and processes by using compost, biofertilizers (BFs), and biocontrol agents (BAs). The use of plant beneficial microorganisms seems to be a very attractive strategy as they are known for their prebiotic, probiotic, and postbiotic functions and as an important part of plant development (Alegria Terrazas et al., 2016; Vassileva et al., 2020a). Recently, the microbial communities associated with the plant have been classified as plant microbiome, which also include viruses, archae, and nematodes (Orozco-Mosqueda et al., 2018). Microorganisms colonize plants creating specific interrelations, including pathogen protection and enhanced nutrient mobilization and acquisition (Lugtenberg and Kamilova, 2009; Gupta et al., 2022). Undoubtedly, these interrelations have been historically affected by the conventional agricultural activities aimed at increasing the yields of crops (Perez-Jaramillo et al., 2015). Introducing microorganisms and organic fertilizers into soil-plant systems is considered an important tool in overcoming problems associated with the excessive use of chemical fertilizers and pesticides (Bashan et al., 2014; Malusà et al., 2021; Shaji et al., 2021). By this reason, there is a strong tendency to stimulate application of microorganism-bearing products to re-establish and enhance soil fertility and crop production and quality particularly in a stressed environment (Shilev et al., 2019).

It should be distinguished between traditional microorganism-bearing fertilizers, such as compost or animal manure and formulated biofertilizers. While in the traditional microorganism-bearing fertilizers, there is a wide range of well-studied and categorized, including pathogenic, microorganisms, which in some cases are difficult to control, BF/biocontrol products normally containing one or more microbial cultures, with guaranteed quality and cell quantity and, in some products, controlled release after introduction into the soil-plant system (Venglovsky et al., 2006; Young et al., 2012; Malusà et al., 2021). It should be noted, however, that there is a discussion in the scientific literature on the potential risk for humans and animals when commercial plant beneficial microbial formulations are introduced into soil-plant systems (Deising et al., 2018). The main concern is that it is difficult to distinguish between plant beneficial and opportunistic pathogenic microorganisms as they have similar properties and characteristics (Berg et al., 2005, 2013; Lu et al., 2021).

Similarly, after application of organic fertilizers, such as compost or animal manure, particularly fresh fruit and vegetables have been repeatedly reported as vehicles of pathogenic microorganisms, such as *Listeria monocytogenes, Staphylococcus aureus* (Johannessen et al., 2002), *Enterococcus faecium, E. faecalis*, *L. monocytogenes* (Johnston et al., 2006), *Salmonella enterica* (Branquinho Bordini et al., 2007), *Escherihia coli* O157:H7 (Beretti and Stuart, 2008), and *E. coli* O104:H4 (Mellmann et al., 2011). In the latter case, the pathogen was found in seeds and caused the hospitalization of over 800 individuals and 53 deaths in Germany, followed by 4 other countries, which indicated the low level of attention to this problem of all players involved in the production chain (including scientific organizations). It is also important to note the negative results from microbiological tests of the suspected seeds (European Food Safety Authority, 2011) and, in general, the difficulty to determine pathogenicity through screening for virulence genes.

The aim of this mini-review was to reveal how safe are the above products, before and after their application following the 3-P approach (prebiotics, probiotics, and postbiotics). During the past years, the analysis of risk factors for both types of microorganism-bearing products is limited to single strains or single product (e.g., commercial organic waste or formulated product) without presenting a global view of all products containing pathogenic or beneficial microorganisms. Special, but limited, attention will be given to some regulatory issues concerning Spain but also those aimed at European harmonization of safety parameters of bio-based products, which are now not homogenous at national and regional levels. In fact, safety issues and measures in the field of microbially bearing fertilizers should be oriented in several main directions: a) before field application while treating organic fertilizers or producing BFs; b) during application of the microbial products; and c) during harvesting operations and postharvesting services. Here, the first two key points are discussed. We consciously do not discuss here the effect of microorganism-bearing products on plant microbiome as interactions between various biotic and abiotic factors in soil-plant systems, host preferences, selection of highly competitive smart microbial consortia, and their suitable formulations for preserving cell viability after storage and delivery need more studies (Orozco-Mosqueda et al., 2018).

## Risks of Traditional Microorganism-Bearing Fertilizers

**FIGURE 1:**
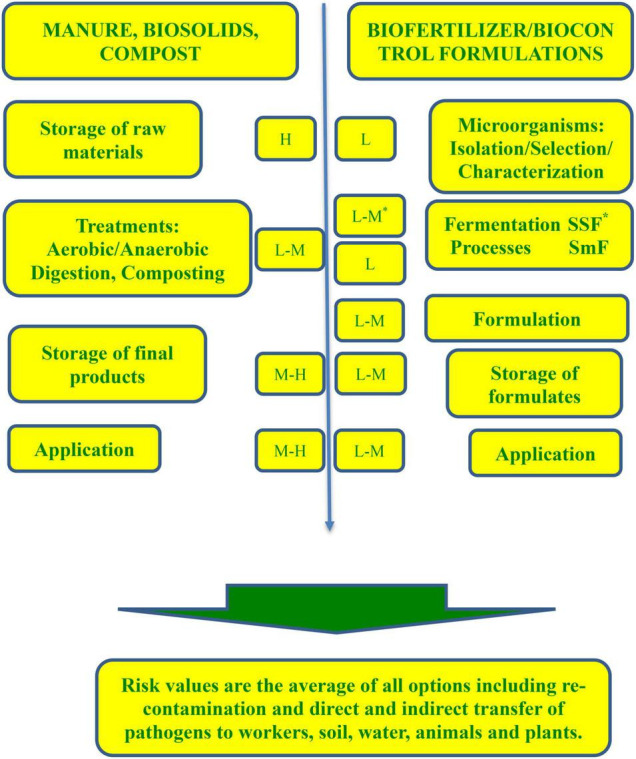
Risks of contamination of microorganism-bearing fertilizer preparations during different stages of their production (L-low risk; M-medium risk; H-high risk).

Prebiotic materials, such as biosolids, animal manure, and compost, alone or combined, are the most applied organic-waste-based fertilizers, as they increase the input of carbon and nutrients to the soil (EIP-AGRI, 2015). However, due to the presence of microorganisms in their composition and despite the official statements in many countries that these products are microbiologically safe before application to soil, the scientific community continuously publishes data or opinions of concerns (refer to Figure 1). The main reason is the difference between the recommended treatment procedures by the authorities and their practical use. For example, in a recent analysis, Ramos et al. (2021) reported that farmers frequently consider the manure aging (storage time needed to reduce the manure pathogenicity) as composting thus introducing live microorganisms existing in the “treated” material into the soil. Moreover, particularly in Spain, illegal or sub-standard landfilling is still widespread practice with all risks of water, air, and soil contamination and potential health problems for animals and humans (EC Country Report Spain, 2019). Since food safety problems provoked by pathogen-contaminated roots or leaves increase (particularly in fresh produce and minimally processed crops), the European Commission investigates such cases strictly and takes measures through well-established procedures.

### Biosolids

Biosolids are “nutrient-rich organic materials resulting from the treatment of domestic sewage in a treatment facility that can be recycled and applied as fertilizer to improve and maintain productive soils and stimulate plant growth” (EPA, 1994). The introduction of sewage sludge (i.e., biosolids) in agricultural soil-plant systems is a subject of criticism, particularly as a source of heavy metals and human pathogens. However, it is also well established that after the treatment of sewage in waste-water treatment plants, the number of pathogenic microorganisms in the effluent is greatly reduced. After analyzing the microbial community structure of several sewage sludges, two clusters of dominant genera were found: one included *Propionibacterium*, *Comamonas*, *Brevundimonas*, *Methylobacterium*, *Stenotrophomonas*, and *Cloacibacterium*, while the other cluster included *Clostridium*, *Treponema*, *Desulfobulbus*, and *Syntrophus* (Nascimento et al., 2018). Sludges revealed high bacterial diversity, but their sources and redox operating conditions, as well as liming, did not consistently affect bacterial community structures. Particularly efficient for the reduction of bacteria, fungi, helminth eggs, and viruses are composting, settling ponds, dewatering drying, and pH elevation (>9) (Adegoke et al., 2016). Nevertheless, biosolid-derived pathogens can easily enter the soil-plant-food chain, which might provoke safety decrease (Makádi et al., 2007; Bastida et al., 2008). In addition, a substantial amount of antibiotic-resistant bacteria (ARB), antibiotics, heavy metals, and antibiotic-resistant genes (ARG) remain after treatment and further invade soil-plant systems and microbial community (Karkman et al., 2018; Nunes et al., 2021). Heavy metals in biosolids and other wastes exist, including after treatment in the form of a molecule or ion, thus ensuring horizontal gene transfer between different bacteria (Ezugworie et al., 2021). Recently, thermally dried anaerobically digested sewage sludge has been proved as a source of ARGs and mobile genetic elements (MGEs), thus increasing the risk of antibiotic resistance dissemination in agricultural soils (Jauregi et al., 2021).

### Manure

Manure is one of the most important organic sources of nutrients, containing microorganisms, and deserves special attention. Livestock manure, according to the last EC Directive, means “waste products excreted by livestock, or a mixture of litter and waste products excreted by livestock, even in processed form” (EU Commission, 1991). Manures are different and, depending on their origin, can be solid, semisolid, and liquid, containing mixtures of feces, urine, bedding materials, including various chemical or physical wastes (Shober and Maguire, 2018). The total production of manure in Europe is 140 million tons on a dry matter basis (Scope Newsletter, 2014), which European farmers, following the European Environmental and Fertilizer policy measures, should process before application in soil (EU Commission, 2013). Manure contains a wide variety of microorganisms (about 10^8^–10^10^ CFU/g), including pathogens, which present health risks for animals or humans. Among viruses, fungi, and bacteria in manures, typical pathogens, including *Salmonella* sp., *E. coli* O157 H7, *Campylobacter jejuni, Yersinia enterocolitica*, and *C. perfringens. Salmonella*, are *Enterobacteriaceae*, which are widely distributed and include more than 2,000 serotypes (Venglovsky et al., 2006) (for detailed microbial characteristics of manure see Black et al., 2021). The initial number and profile of pathogens in manures and their characteristics change as a function of the manure type, storage conditions, and after treatment (Hutchison et al., 2005). The EC legislation permits the use of all types of manure except “factory farming” manures (EC Regulation 889, 2008), and in many countries, the application of liquid manure is not allowed. Therefore, it is important to develop and select strategies to manage not only the nutrient content in manure without affecting soil, water, and air but also reduce pathogenic microorganisms by microbiological, chemical, or physical methods (Heinonen-Tanski et al., 2006). In general, most enteric microorganisms do not multiply and survive out of the host due to the enormous stress conditions and after a long period of storage, but early studies reported that low temperatures and high solid concentrations increase the probability of survival of many pathogens (Feachem et al., 1983; Kudva et al., 1998). For these reasons, and particularly in case of factory farming, it is recommended to clean manure, normally by biological or thermal treatment (Venglovsky et al., 2006). In any case, management of manure during storage and further treatment (e.g., by composting) and its proximity to plants from one side, and the always existing possibility of runoff, formation of contaminated dust, and animal movements from the manure storage to crop fields, on the other side, should be an important consideration when analyzing all safety risks on farm sites (Ramos et al., 2021).

### Composting

Composting is the biological process of choice for manure and sludge treatment as it is cost-effective and decreases pathogenic bacteria, fungi, and helminthic eggs, thus producing high-quality products enriched with humic acids. Slurry and sludge treatment may include aerobic stabilization when the temperature rises to 70°C thus becoming free of pathogens (Soares et al., 1995). It was reported that in composting reactors or aerated static-piles at a temperature of up to 60°C/3 days and following the existing regulations, pathogen destruction reached the most probable number of 1,000 coliforms/g dried solids and ≤ 3 salmonellae/4 g dried solids, but these processes should be well controlled as materials that are treated many times do not meet these standards, thus allowing pathogen regrowth.

It seems impossible to achieve organic fertilizers and ambient surrounding the storage/treatment facilities totally free of pathogenic or potentially pathogenic microorganisms. Enteric viruses and *Salmonella* spp. were found in liquid manure after the anaerobic bio-digestion process (Fongaro et al., 2014). It has been also reported that viable bacteria and viruses immobilized on air-dust particles have a greater ability to survive and affect human and animal health (Takai et al., 1998). Bio-aerosol, containing mainly Firmicutes and Actinobacteria, is continuously released starting with the pretreatment of raw materials and during the open-air process of composting without any form of control (Wery, 2014). Around the composting facilities, the bacterial diversity reaches 1.5–15.3% bacterial cells, but other microorganisms, including *Aspergillus* and *Penicillium*, can also be found in the air (Walser et al., 2015). The selection of specific raw materials and effective process management produced different levels of bacterial and fungal diversity (Hernández-Lara et al., 2022). Therefore, the risk of biological contamination of farmworkers is high, and safety measures should be continuously improved (OECD, 2012). Approaches related to determining the number of cells/spores in environments surrounding production facilities thus monitoring, for example, airborne particles could be included in disease management systems when working with organic residues (Mahafee, 2014).

Another point of attention, which was mentioned in the manure part of the mini review, concerns antibiotic resistance in soil (Wang and Tiedje, 2020). Particularly intensive are studies on manure as a vehicle of ARGs, which, once transmitted, potentially are a great risk to public health. Moreover, MGEs boost the horizontal gene transfer of ARGs in the environmental microorganism. It was found that the wide application of animal manures in organic agriculture inevitably enriches the already existing ARGs pool in soils but also additionally introduces exogenous ARGs, which can be found in soil for a period ranging from few weeks to several months depending on the manure and soil characteristics. There are a wide number of studies on the mechanisms and interactions in manure- or compost-enriched soil, which try to explain the regulation or control of the persistence of ARGs in soil for different periods of time, but the fate of ARGs where manure from different sources has been repeatedly implemented is not fully understood although some ARGs could be found in deep soil carried by their host bacteria (Li et al., 2022).

## Biofertilizer and Biocontrol Products, Pathogen-Free or Pathogen Stimulating?

A biofertilizer can be defined as the formulated product containing one or more microorganisms that enhance the nutrient status (and the growth and yield) of the plants by either replacing soil nutrients and/or by making nutrients more available to plants and/or by increasing plant access to nutrients (Malusa and Vassilev, 2014). BAs can be defined as living organisms or natural products derived from living organisms, including microorganisms, that are used to suppress plant pathogen pest populations (Panpatte et al., 2016). All these products are based on the activity of one or more microorganisms and can be commercialized in liquid or granular form (Bashan et al., 2014). Contamination of the BF/BA could be observed in the production/formulation stage as well as during the storage. In fact, contamination is one of the main reasons for unsuccessful field application of plant beneficial microorganisms; an early study by Herrmann et al. (2013) demonstrated that 37% of the tested formulated products could be considered as “pure”; 63% were contaminated with bacteria and 40% contained only contaminants.

The schemes of selection, production, and formulation of microbial plant beneficial products are well developed (Vassilev et al., 2001a,^2015^, 2017a; Malusá et al., 2012; Bashan et al., 2014; Vassilev and Mendes, 2018; Vassileva et al., 2020a). Usually, at least one, two, or three different microbiome members can be included in the final product (Vassilev et al., 2001b,c,d, 2020; Sahu and Brahmaprakash, 2016). All operations starting from the inoculum preparation, fermentation process, and downstream stage, including the product formulation as well as packaging, are carried out in sterile conditions, and, therefore, these biotechnological products should be free of contaminants. The production of spores, biomass, or metabolites is normally carried out in closed liquid submerged (e.g., batch and fed-batch) or solid-state fermentation systems with well-controlled parameters and improved quality of the final product (Vassileva et al., 2021). However, there is a risk of biological contamination in each one of the production process stages deriving from water, air, equipment, nutrient media, and laboratory/plant technical staff. The starter inoculum should be carefully managed to avoid contamination, mutation, and phenotypic changes during the fermentation process that may result in the production of ineffective BF or postbiotic (i.e., metabolic) biostimulants with different characteristics (Nims and Price, 2017). Contamination is also possible during the formulation stage or after introduction into the soil. For example, two endophytic fungi (i.e., *Muscodor albus* and *M. roseus*) producing volatile myco-fumigants were formulated in a mixture of water-absorbent starch, corn oil, sucrose, and fumed silica (Stinson et al., 2003). The produced formulations reduced the disease incidence of soilborne pathogens, but plant growth reduction was observed due to the growth of deleterious rhizobacteria on some components of the complex carrier.

### Is There Any Risk of Pathogen Contamination in the Chain “Plant Beneficial Microbial Products-Soil/Plant/Food/Humans/Animals”?

During the past years, serious doubts appeared in the security and safety of plant beneficial microorganisms. As mentioned above, Deising et al. (2018) suggested possible changes in microbial community profile and appearance of secondary metabolites, such as aflatoxin, ochratoxin, patulin, and mycotoxins, after the introduction of plant beneficial microorganisms in soil-plant systems. It is interesting to note, in this sense, that risk assessment of plant beneficial microorganisms is not included in the corresponding legislations although many plant growth- and health-stimulating microorganisms are suggested as opportunistic human pathogens (Berg et al., 2005). The dual behavior of soil microorganisms was frequently described, thus increasing the need for serious preliminary testing. For example, *Aspergillus terreus*, known as both the plant growth stimulator and BA, produced terrain, one of the numerous genome-analyzed secondary metabolites released by *A. terreus* (Gressler et al., 2015). Terrain inhibits seed germination and plant growth, provokes plant surfaces’ damage, and inhibits the growth of competitors, thus facilitating the fungal invasion in the respective environmental niche (Vassileva et al., 2020b). When introduced into the human body, like other members of the genus *Aspergillus*, *A. terreus* can cause aspergillosis infection with a high level of mortality particularly in immunocompromised persons (Bartash et al., 2017). Similarly, *Stenotrophomonas* are present in manure samples and particularly *S. rhizophila*, after physiological and molecular studies, are found safe and have a high plant beneficial potential without human pathogenic traits. Recent studies propose *S. rhizophila* as a promising PGP and biocontrol product. However, some *Stenotrophomonas* species demonstrated dual characteristics, promoting plant growth and health with a simultaneous multidrug resistance affecting immunosuppressed patients, which was further confirmed by a genome analysis (Denton et al., 1998). It should be noted the bioaerosol concentration during the biostabilization of sewage sludge ranged from 160 to 1,440 cell/m^3^, and species, such as *S. rhizophila* and *Fusarium graminerum*, with high bioaerosolization indexes that could be threats to human health were recently identified (Lu et al., 2021). Other microbial strains belonging to the most studied and commercially available genera *Pseudomonas, Enterobacter, Serratia*, and *Burkholderia*, among others, are also known to colonize both plants and humans and should be tested at least before starting serious biotechnological experimental work on their mass production, formulation, and application (Zachow et al., 2009). A well-studied case is that of the *Burkholderia cepacia* complex, a group of phenotypically associated bacterial species that have known PGP traits, including N_2_ fixation, but can also be opportunistic human pathogens (Eberl and Vandamme, 2016). Another intensively debated bacterial genus is *Pseudomonas*, which encompasses several PGP species (e.g., *P. fluorescens, P. putida, P. putrefaciens*, *P. stutzeri*, and *P. pseudoalcaligenes*) but also the pathogenic species *P. aeruginosa*, an opportunistic pathogen causing respiratory tract infections in humans (Mendes et al., 2013).

Therefore, there are two well-defined tendencies related with the potential pathogenicity of plant beneficial microorganisms. The first one proposes more control and a risk assessment test of all microorganisms before their commercialization, while the second does not see any reason for concerns about their safety and changes in the actual registration rules (Koch et al., 2018). Which point should the authorities rely on when defining the safety measures for workers dealing with the production and application of manure, compost, and BFs? In the latter case, the new EC Regulation, which is foreseen to start in 2022, states that a microbial plant biostimulant should be reduced to mycorrhizal fungi, *Azotobacter* spp., *Azospirillum* spp., and *Rhizobium* spp., thus limiting the possibility of introducing opportunistic pathogens into the food chain. In contrast, from farm-workers-safety point of view, the formulation form (e.g., liquid or solid) of all products containing plant beneficial microorganisms is important. In case of gel-based formulations, immobilized cells are embedded in a polysaccharide matrix (Vassilev and Vassileva, 2005), thus reducing the risk of direct contact between cells and workers handling the product although in this scheme, a potential plant-soil contamination is possible. For example, additives included into the carrier matrix (Vassilev et al., 2020) could attract other microorganisms, including pathogenic strains, during storage or transportation. However, to avoid contamination, carriers, such as k-carrageenan, can be used, which after drying reduce their volume and water content. For these reasons, it is recommendable to do a thorough risk assessment of all these amendments for environment-animals-humans before the process of registration regardless of their risk group and plant beneficial effects.

## Microbially Based Fertilizers’ Assessment and Safety Measures

When analyzing the above information, two modes of contamination by plant beneficial microorganism-bearing products can be distinguished, namely, (1) direct contamination of people working on their production and application and (2) contamination of mainly fresh production grown in environment contaminated by these products with further effects on consumers.

As recently summarized by the COST Action SACURIMA (Leppälä et al., 2021), the European Community described high rates of injury, occupational disease, and exposures in Agriculture. Each year, there are about 6 reported accidents per 100 workers and 12 reported fatal accidents per 100,000 workers in Agriculture (Eurostat, 2019). Over 40% of agricultural workers feel unsafe at work. Over 15% report exposure to skin and respiratory diseases. About 4% suffer from work-induced respiratory illnesses. In addition, foreign workers (mainly migrants) have a higher risk for occupational injuries than native workers (Casey et al., 2015). Bearing the above in mind, we should first assess how the fertilizers containing microorganisms affects the manpower.

**FIGURE 2:**
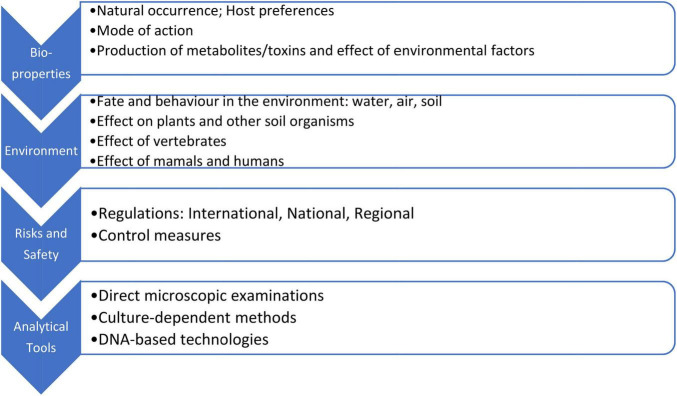
Analytical tools and measures for characterization and monitoring of microorganisms before and after their release in the environment.

Management of safety measures in both preparation and application of microorganism-bearing organic fertilizers and biotechnologically produced BFs/biocontrol formulates is based on the understanding of how microorganisms from microbially-derived products or their derivatives could reach and enter the human body (Figure 2). As a rule, it is not possible to know if a given microorganism is completely “safe.” Therefore, the general measure is to avoid contact with microorganisms and their metabolites. One of the most important actions to follow includes assessing and avoiding all possibilities of inhalation of contaminant’s aerosols and airborne particles (as mentioned earlier) and contamination of hands, eye-hand contact, or absorption through intact skin (Liberman, 1984; Peng et al., 2018). In contrast, some producers of BFs apply techniques, which are far from the biotechnological normal and safe procedures, using open-air fermenter-like vessels, or inoculate microorganisms directly to open-air storage reservoirs of waste substrates (corresponding author information), thus allowing growth and regrowth of both beneficial and pathogenic microorganisms (including spore-forming). Similar regrowth can be observed in composting processes when *Salmonella* spp., *E. coli*, and *Listeria* sp. are present in not-matured composts. All these and similar practices should be controlled and forbidden.

In general, the concept of biosafety is applied first to ensure the safe work with pathogenic microorganisms in the laboratory. Starting from the production of BFs and BAs, it should be mentioned that in biotechnological laboratories and plants, there are many operations, such as centrifugation, homogenization, mixing, blending, aeration of liquids, release of liquids under pressure, and handling of solids, which can form highly contaminated aerosols (Lelieveld et al., 1995). Similar is the contact with biological substances, which occurs during the handling of manure and other organic materials before and after composting due to exposure to liquid/solid particles containing microorganisms or microbial metabolic products. The most frequent diseases in the agricultural sector by biological agents are provoked by bacteria, fungi, and viruses that enter the body through the respiratory, dermal, or digestive routes: Allergies or sensitization processes, such as the farmer workers’ lung; Aspergillosis; toxic organic dust syndrome due to worker exposure mainly by inhalation of microbially derived proteins and toxins; and carcinogenic, mutagenic, immuno-toxic, neuro-toxic, hemato-toxic, and hormonal disorders caused by filamentous fungi, such as *Aspergillus*, *Fusarium*, and *Penicillium* producing mycotoxins.

One of the main reasons is the difficulty of controlling the spread of various pathogenic microorganisms, which are resistant to high temperatures, such as fungi and/or sporulating bacteria, before and after treatment in plants and during storage (EU-OSHA, 2009). As a special case/option, it could be mentioned that a microbially treated organic matter enriched with plant beneficial *A. niger* and solubilized P could be partially incinerated at 350–500°C to reduce its volume and, consequently, increase P concentration but also to avoid the presence of microbial biomass/spores (Mendes et al., 2015).

In the production, storage, and application of formulated BF and biocontrol products, workers and biotechnologists should follow the Good Microbiological Practice (GMP) rules, which are normally in use when working with low-risk microorganisms belonging to class I (i.e., non-pathogenic microorganisms). This set of rules is developed to prevent laboratory workers from exposure to the microorganisms and, simultaneously, prevent microorganisms from environmental factors, including contamination by other biological material; it is known that 80% of lab operators carry mycoplasma, which is highly infectious and changes cell metabolism and growth (Falkow, 2008). One of the most important points of the safeguarding strategies should be the information of lab/bio-plant workers and farmers: information, in sense of education and communication, as suggested by the Final Report of the EIP-AGRI (European Innovation Partnership Agricultural Productivity and Sustainability) focus group on soil organic matter in Mediterranean regions (EIP-AGRI, 2015). They should be well informed about the production details, product quality, product composition, microbial content, potential microbial pathogens and their effects on human health, protection measures, mode of product action in soil-plant systems, and advantages of product application. Authorities and producers should inform farmers on the maximum permitted and real number of pathogens. For example, the Spanish Directive on Fertilizers (Real Decreto 506/2013) fixes the maximum amount (CFU) of the most abundant pathogens in traditional microorganism-bearing organic fertilizers, *Salmonella* and *Escherichia coli*, what is in accordance with the Proposal for a European Commission, 2009. However, information on other potentially pathogenic microorganisms in organic fertilizers is missing. In the recent EC Regulation (Regulation EU 2019/1009 of the European Parliament and of the Council, 2019), organic and organo-mineral fertilizers should be free of *Salmonella* and *Escherichia coli*. Additionally, plant biostimulants should be free of *L. monocytogenes, Vibrio* spp., *Shigella* spp., and *S. aureus*, while for *Enterococcaceae*, anaerobic microorganisms, yeasts, and molds, the CFU can be 10, 105, and 1,000 per g or ml. To fulfill these requirements, and particularly in case of organic fertilizers, all crude materials should be treated in special installations (e.g., plants). Therefore, farmers and workers should be informed about the whole production process starting with the initial operations of recollection and ending with the application of the commercial products and in contrast, correspondingly equipped.

A recently published review questioned the whole process of production and application of microbially based fertilizers and the basic understanding on what pathogenicity is (Hardoim et al., 2015). In contrast, the wide ecological and metabolic diversity in microbes, their relatively short generation time, and their ability to rapidly adapt to and colonize highly specific niches, including specific compartments of animals, humans, and plants, allow some microorganisms to cause disease. For this reason, in the new EU Regulation, the label of potentially dangerous microbial fertilizers (i.e., biostimulants) shall contain the following phrase: “Microorganisms may have the potential to provoke sensitizing reactions” (Regulation EU 2019/1009 of the European Parliament and of the Council, 2019). Special attention and measures need the presence of antibiotics in the microorganism-bearing natural organic amendments and their antibacterial activity, which could affect the structure and function of natural microbial communities thus promoting the accumulation in soil of antibiotic-resistant bacteria ARB and ARGs. The recently adopted EU Commission, 2019/6 on veterinary medicinal products requires that any risk associated with the development of antimicrobial resistance (AMR) must be considered (EU Commission, 2019). However, there is no generally accepted approach for assessing the risk of development or dissemination of AMR in the environment. This problem could be easily solved if antibiotics use is controlled every day individually based on the administration history and the farm environment is strictly managed (Suzuki et al., 2022).

Field workers dealing with potentially dangerous plant beneficial products containing microorganisms could be divided into groups according to the tasks: general tasks, including work with soil, seeds, planting, watering, treating plants with chemical and organic fertilizers, and other groups working directly with animals and derived wastes or working with microorganisms (in case of Spain: Instituto Nacional de Seguridad e Higiene en el Trabajo, 2014). Each group has its specific norms, National but also local Directives prepared by each Spanish province with instructions on how to work with materials contaminated by or bearing microorganisms and how to react in case of emergencies. Therefore, this part of the safety measures is administratively/normatively well-organized for the local workers. However, with the recent migrants’ entrance in many EC countries, including Spain, some of them are introduced into diverse agricultural activities thus changing the traditional workforce profile. These newcomers will need special attention as inadequate and unskilled, inexperienced human resources may be easily subjected to microbial contamination when working with microbially bearing products (Itelima et al., 2018). In any case, authorities, biotech producers, and farm workers should be prepared to rapidly analyze scientific elaborated risk assessments of microbially bearing fertilizers concerning human, animal or plant health, safety, or the environment (for more detailed information regarding regulations in the field of BFs, refer to Malusa and Vassilev, 2014).

There is another very important issue when assessing all potential risks of applying microorganism-bearing materials as soil amendments thus potentially entering determined strains in the plant/human/animal microbiome. A recent study analyzed the intime role of microorganisms in mechanisms of development and progression of cancer (Parhi et al., 2020). Microbial species, including *Streptococcus gallolyticus*, *Enterococcus faecalis*, enterotoxigenic *B. fragilis*, enteropathogenic *Escherichia coli*, and *Fusobacterium* spp., were registered in these studies. A typical example is the bacterium *Helicobacter pylori*, which increases the risk of cancer in the stomach. Enterococci are part of the intestinal microbiota in a great variety of hosts. They are particularly abundant in feces of warm-blooded animals and demonstrated a long-term survival in the environment (Wasteson et al., 2020). The question that appears is: Is there any risk of contamination with microorganisms resistant to treatments found in human and animal cancers through sewage sludge/manure/composts applied in the soil-plant-food chain?

## Conclusion

The main conclusion that could be formulated at the end of this short mini-review is the need of well-regulated and controlled circulation of microorganisms in agricultural ecosystems with further health-beneficial effects on consumers. This approach could ensure highly efficient and safe microbially based and chemicals-free sustainable agriculture. Better structured safety assessment and risk management measures should be developed based on existing knowledge of the microorganisms. From safety point of view, the first reason of concern is the nature, characteristics, and mode of treatment or production of fertilizer with microorganisms in its composition. Manure and sewage sludge are the main natural sources of potential risks, but serious safety measures are foreseen at the European and National levels. Incentive actions are offered to reduce their potential field application while enhancing alternative uses in composting plants or energy generation and further use of the resulting products (digestates, biochar, etc.). Plant microbiome consists not only of beneficial but also pathogenic microorganisms. Therefore, another important point is the fate of the introduced microorganism-bearing fertilizers, particularly biostimulants, in soil and their effect on the microbial community structure, including autochthonous soil pathogens. In general, we should know the ecological behavior of introduced microorganisms, and the possibility of interactions must be considered in risk assessment actions (Malusà et al., 2021). As suggested by van Elsas et al. (2012), soil microbial diversity and the level of its metabolic activity is the key regulating the fate of a given microorganism introduced in soil. Soil biodiversity dynamics is a multidirectional process where soil management, applied microbial biostimulants, and organic matter interact with the autochthonous microflora within a functioning ecosystem (Vassilev et al., 2021). In short term, the introduction of nutrients derived from the organic fertilizer (compost, treated manure, etc.) or metabolites released from introduced microbial biostimulants might stimulate the growth of whichever microorganism from the plant microbiome, including plant-associated pathogens (Berg, 2009). Therefore, microbial toxicological data, metabolite profile in field conditions, and long-term experiments on assessing the risk for the environment should be performed. Particularly important are risk studies and determination of potentially invasive fungi and bacteria able to survive in stress conditions (Vassilev et al., 2012; Alavi et al., 2014).

Another important conclusion of this short analysis of the development of measures for safe production and use of microbial-based fertilizers is that this field of research and biotechnological/agronomical activity needs a strict but flexible legal framework based on the available database to further support the transition toward more sustainable agriculture. In this sense, a better methodological approach is needed to determine the potential pathogenic power of the plant beneficial microorganisms before their direct industrial production and formulation. In a recent article, Vílchez et al. (2016) proposed a simple, cheap, and efficient strategy to evaluate the potential risk of plant growth-promoting microorganisms for human, animal, and plant health, avoiding the use of vertebrate animals. Another important approach could be the limited use of potentially pathogenic organic materials and their substitution by carefully risk-tested beneficial microorganisms combined with organic matter used in their production process. Manure could be applied after more strict treatments, while compost could be enriched with safe beneficial microorganisms. Although many basics of microbiome biology remain unresolved, could we manipulate the animal microbiome through animal breeding and dietary control thus preventing the presence of pathogens in the manure (Huws et al., 2018)? In contrast, following the principle of One Health approach, microorganisms derived from human gut microbiota can be considered in the near future as a PGP-biocontrol option. By applying this scheme, we could produce safe fresh products rich in probiotics. Thus, the natural circle of soil-plants-humans-soil could be reactivated. In some cases, plant beneficial metabolites (postbiotics) could be applied instead of their producers (Mendes et al., 2017; Vassilev et al., 2017b), thus avoiding the direct application of microbial cells but assessing the effect of all microbial metabolites.

## Author Contributions

MV and NV designed and drafted the work. SM, LC, EM, LG, VM, and EF-P contributed to the revision of the manuscript. All authors contributed to the article and approved the submitted version.

## Conflict of Interest

The authors declare that the research was conducted in the absence of any commercial or financial relationships that could be construed as a potential conflict of interest.

## Publisher’s Note

All claims expressed in this article are solely those of the authors and do not necessarily represent those of their affiliated organizations, or those of the publisher, the editors and the reviewers. Any product that may be evaluated in this article, or claim that may be made by its manufacturer, is not guaranteed or endorsed by the publisher.
